# “Are Thai children and youth sufficiently active? prevalence and correlates of physical activity from a nationally representative cross-sectional study”

**DOI:** 10.1186/s12966-017-0529-4

**Published:** 2017-05-30

**Authors:** Areekul Amornsriwatanakul, Leanne Lester, Fiona C. Bull, Michael Rosenberg

**Affiliations:** 1 School of Earth and Environment and School of Sport Science, Exercise and Health, University of Western Australia, Perth, Australia; 2 School of Sport Science, Exercise and Health, University of Western Australia, Perth, Australia; 3 Centre for Built Environment and Health, School of Earth and Environment, University of Western Australia, Perth, Australia

**Keywords:** Children, Youth, Physical activity, Sport and exercise, Prevalence, Correlates, National survey, Epidemiology, Thailand, NCDs, And national policy

## Abstract

**Background:**

Children and youth gain multiple health benefits from regular participation in physical activity (PA). However, in Thailand there is limited national data on children and youth’s PA behaviors and recent reports suggest that Thai children and youth have low levels of PA. Furthermore, there is almost no data on the factors associated with inactivity to support the development of a Thai National PA Plan. The purpose of this paper is to investigate Thai children and youth’s participation in PA and its correlates across sociodemographic characteristics and different PA domains.

**Methods:**

This study applied a cross-sectional study design with a multi-stage stratified cluster sampling. A national representative sample of 13,255 children and youth aged 6-17 years were used for data analysis. A previously validated questionnaire was used to assess PA prevalence. Logistic regression was conducted to examine the relationships of socio-demographic factors, and participation in different PA domains with overall PA.

**Results:**

Only 23.4% of Thai children and youth met recommended levels of PA and there were large gender and regional differences. PA levels generally declined with age, although the level observed in the 10-13 year group was slightly higher than other year groups. A majority of children and youth engaged in a large number of different activities across PA domains. Sex, age, BMI, geographical regions, organized sports, participation in sport and recreational activities were significant predictors of meeting the global PA guidelines, whereas participation in physical education, active transport, and the number of screen time activities had no association. Girls were less likely to achieve sufficient PA levels (OR = 0.49, 95%CI; 0.45-0.54, *p* < 0.001), as were obese children (OR = 0.78, 95%CI; 0.64-0.94, *p* = 0.01), children living in the West (OR = 0.47, 95%CI; 0.38-0.59, *p* < 0.001), and those who did no participation in organized sports and sport/exercise activities, or minimal participation (1-2 activities) in recreational activities (OR = 0.79, 95%CI; 0.68-0.90, *p* < 0.001).

**Conclusions:**

The prevalence estimate of meeting the recommended guideline of sufficient PA in Thai children and youth is low, despite the high levels of engagement in a large number of PA. The results indicate that policy and interventions aimed at increasing PA are needed with special attention required to address specific groups less likely to meet the PA guideline. Strategies to promote a large volume of participation in all possible types of PA as part of Thai children and youth’s daily life should be considered.

## Background

Regular participation in physical activity (PA) has multiple benefits for children and youth, including enhancement in physical, social and psychological health [1–3]. The World Health Organization (WHO) recommends children and youth (5–17 years old) engage daily in at least 60 min of moderate-to-vigorous physical activity (MVPA) to maintain a healthy cardiorespiratory and metabolic risk profile [4]. However, the majority of children across the globe are insufficiently active [5, 6], with only one in every five 13-15 year olds meeting the WHO guideline [7]. Disparities between countries exist with 15.9% of American youth aged 14-17 years achieving the WHO recommended guideline [8] and 7% of Canadian children and youth aged 6-19 years completing 60 min of MVPA on at least 6 days a week [9]. In Asia, 5.6% of Chinese children and youth aged 9-17 years met the WHO recommended guideline [10]. In Hong Kong, Taiwan, and South Korea, a low proportion (ranging from 13 to 30%) of school-age children participates in a minimum of 30-min-MVPA on 3 days a week [6].

Although global PA guidelines were launched in 2010 [4], Thailand has no agreed baseline on the levels of PA participation in children and youth. There has been a variety of different measurement tools and indicators used, some of which were not designed for use with children. Available data provide limited basis for consensus with prevalence estimates ranging from as low as 12% up to 61% [11–13]. A recent national survey on PA in Thai children and youth reported that 23% of them met the global PA guideline [14] and this is more similar to international trends [15–17]. Similar patterns between sex were observed with Thai boys (29%) participating at greater levels than girls (17%) and the oldest age group reported the lowest prevalence (20%) of meeting the guidelines as seen in other countries [8, 16, 18–20].

However, the recent survey did not provide any information about common PA correlates such as age, sex, and Body Mass Index (BMI). In Thailand, there is limited evidence on the individual, social, and cultural determinants of participation in PA. For example, the relationship between BMI and PA in children and youth is unknown. Internationally, the relationship between these two variables remains inconclusive with some studies suggesting a negative relationship [21–23] and others no relationship [10, 15, 24]. Similarly, within-country geographical differences that predict participation in PA are also unknown making any resource allocation to promote PA at regional level difficult. Besides socio-demographic factors, data on children’s participation in different PA domains including physical education (PE), organized sports, active recreation, and active transport which have potential for policy and interventions are largely unknown in Thailand. Furthermore, the slightly negative relationship between screen time and PA has been identified in many countries [25] but remains undisclosed in Thailand.

The gaps in knowledge and evidence hinder the ability of Thailand to scale up national promotion of PA. For Thailand to be able to develop an effective National PA Plan, data gathered amongst Thai children and youth will provide better evidence rather than relying solely upon data or trends from others, largely western countries. Therefore, the purpose of this study is to investigate Thai children and youth’s PA behaviours, patterns, and correlates of participation across sociodemographic characteristics, and different PA domains.

## Methods

### Setting

Thailand has a population of 66 million across an area of 514,000 sq.km. [26] and is classified as an upper-middle income country, with GDP per capita of USD5,977.4 [27]. Thailand is a free market economy, dominated by manufacturing rather than agriculture [26]. Thailand’s basic education system requires children to spend 6 years completing primary school (grade 1-6), 3 years in junior secondary school (grade 1-3), and 3 years in senior secondary school (grade 4-6) [28]. Overall, Thai children remain in the basic school system between 6-17 years of age.

### Sampling and participants

The Ministry of Education reported that there were 11.1 million students aged 6-17 years attending school across the country in 2011 [29]. A multi-stage stratified cluster sampling was applied to recruit students into the survey. Details of the sampling conducted at each stage are reported elsewhere [14]. In total, 16,843 children and youth from 336 schools in 27 provinces from 9 regions across the country including Bangkok participated in the survey. This sample size is adequate to detect a prevalence estimate of 50% achieving sufficient PA with a confidence level at 90% and 5% precision. Due to the adoption of active school and passive parental consent, student response rate was reflected in the school response rate which was 84.3%. Schools that declined to participate were randomly replaced. Students were provided with an opportunity to decline participation in the survey but there were a negligible number who took this option.

### Data collection

Regional research staff were recruited and trained to administer surveys in each age group and measure weight and height of each student through standard protocols using weight scales and stadiometers brought by the research staff. A data collection protocol was developed for different age groups to accommodate the differences in student capability and maturity. For the 6-9 year group, face-to-face interviews with one to three students were conducted within the school class time. Interviewers used show cards to assist student responses that were recorded by the interviewer. For older age groups (10-13 and 14-17 years), students themselves completed the survey in a classroom setting assisted by three research staff and a class teacher. Data collection in all regions was conducted concurrently from June - August 2015.

### Measures

A self-reported Student Questionnaires developed for Thailand Physical Activity Children Survey (TPACS-SQ) were used to collect data on participation in PA across key domains including PA, sports/exercise, recreation, travel to school, and PE. Socio-demographic, social, psychological and environmental data were also collected. Three versions of the questionnaire were developed by modifying a previously validated instrument which also has three versions for children and youth at different ages, the Child and Adolescent PA and Nutrition Survey (CAPANS) [30, 31]. Validity of the original items used in CAPANS correlated significantly (*r* = 0.40, *P* < .001) with accelerometer data [32]. The level of details in each questionnaire version was tailored to suit the capabilities of the three age groups. The questionnaire version for the youngest age group (6-9 years) was the simplest and excluded questions about frequency and amount of time spending in PA because children at these ages may have difficulties in recalling these information which can be largely variable [33]. The TPACS-SQ was translated into Thai language, pilot tested, and improved before conducting a reliability test on two occasions 3 days apart in the same convenient sample of classrooms. Results from the test-retest analysis showed that the items measuring key variables in the three versions of the questionnaire have sufficient reliability (ICC = 0.34-0.85, K = 0.15-1, % agreement = 33.3-100%). Generally, the Kappa values and the ICC were stronger in older age group, compared to the younger one.

### Measurement of physical activity

*Overall PA participation* was assessed by a single item asking “over the last seven days, how many days were you physically active for a combined total of at least 60 minutes per day”. Children who achieved a total of 60 min of PA on all 7 days were classified as meeting the WHO PA guideline. *Sport and exercise, recreational, and screen-time activities* were measured by providing a list of the activities children might do in the past 7 days with either “Yes” or “No” option. Participation in these activities was categorized into none, 1-2 activities, 3-4 activities, and ≥5 activities. *Active transport* was assessed by asking how children usually travel to school (e.g. by walking, and by bus) and then classifying transport into either “active” or “inactive”. Participation in *PE* was assessed by asking if children had any PE class during the current semester. Participation in *organized sports* was determined by asking if children actively participated in any sports or sport competitions organized by school or any other organizations (excluding PE class) in the last academic year. Participation in PE class and organized sports were categorized into “Yes” or “No”.

### Anthropometry

BMI was calculated based on weight (kg) divided by height (m) square. Children’s BMI were categorized into 4 groups (“underweight”, “normal”, “overweight”, and “obese”) according to the international childhood BMI unofficial Asian cut-offs [34].

### Data management and analysis

All completed questionnaires were collated and data entry was conducted by a trained group of research staff. Data were double entered in CSPro V6.1 (U.S. Census Bureau) and manual checks were undertaken to rectify discrepancies. Final datasets from each region were centrally collated and systematically cleaned. After removal of ineligible cases and cases with missing data on any key variables (21%), a total of 13,255 student surveys were included for analysis. All data were weighted against age, sex, and regional distributions provided by the Ministry of Education [35]. Descriptive statistics in SPSS v 23 were generated to describe sample characteristics and prevalence estimates of PA. Chi-square tests were performed to examine gender differences in sample characteristics, proportions of children meeting the PA guideline between regions, and PA domains. Associations between participation in different PA domains and the achievement of the PA guideline were also examined using Chi-square statistics. Logistic regression was conducted to explore socio-demographic predictors of children achieving the PA guideline.

## Results

Table 1 shows the frequency distribution of boys and girls participating in the survey by age group, BMI, religion, and geographical regions (Upper and lower North were collapsed into North, and upper and lower South were collapsed into South). Similar proportions of boys (51%) and girls (49%) participated in the survey. A majority of children and youth were Buddhist (94%) and underweight (53%). There was no significant difference in gender between age groups, religion, and geographical regions (all *p* > 0.05). A significantly greater proportion of girls were within the normal BMI category than boys (X^2^ = 24.83, *p* < 0.001).

**Table 1:** Characteristics of the samples

n (%)	Total (*n* = 13,255)	Boy (*n* = 6718)	Girl (*n* = 6537)
Age group			
6-9 years old	4274 (32.2)	2189 (32.6)	2085 (31.9)
10-13 years old	4486 (33.8)	2271 (33.8)	2215 (33.9)
14-17 years old	4495 (33.9)	2258 (33.6)	2237 (34.2)
BMI*			
Underweight	7102 (53.6)	3645 (54.3)	3457 (52.9)
Normal	3877 (29.2)	1855 (27.6)	2022 (30.9)
Overweight	1353 (10.2)	701 (10.4)	652 (10.0)
Obese	923 (7)	517 (7.7)	406 (6.2)
Religion			
Buddhism	12,456 (94.0)	6321 (94.1)	6135 (93.9)
Islamic	674 (5.1)	338 (5.0)	336 (5.1)
Christian	125 (0.9)	59 (0.9)	66 (1.0)
Geographical region			
Bangkok	726 (5.5)	364 (5.4)	362 (5.5)
Central	1576 (11.9)	802(11.9)	774 (11.8)
East	1280 (9.7)	658 (9.8)	622 (9.5)
West	1249 (9.4)	646 (9.6)	603 (9.2)
North	2934 (22.1)	1445 (21.5)	1489 (22.8)
South	2885 (21.8)	1446 (21.5)	1439 (22.0)
North East	2605 (19.7)	1357 (20.2)	1248 (19.1)

*Significant difference between genders at *p* < .001

^a^Percentages may not add to 100% due to rounding

Overall, 23.4% of Thai children and youth met the PA guideline of at least 60 min of PA everyday (Table 2). The proportion of boys who met the PA guideline was almost twice that of girls (Boys: 30.1%; Girls: 16.8%). The proportion of girls meeting the PA guideline was significantly less than boys in all age groups and regions (*p* < 0.001). The prevalence of meeting the PA guidelines was highest in children aged 10-13 years (26.3%), followed by children aged 6-9 years (24.4%) and 14-17 years (19.4%). The prevalence estimates at the regional level ranged from 16.0% in the West to 26.7% in Bangkok. Children’s sex (X^2^ = 326.38; df = 1; *p* < 0.001), age group (X^2^ = 65.63; df = 2; *p* < 0.001), and region (X^2^ = 75.23; df = 6; *p* < 0.001) were strongly associated with meeting the PA guideline.

**Table 2:** Percentage of Thai children and youth who met physical activity guideline by region, age group, and sex

Region	Total (*n* = 13254*)		6-9 years (*n* = 4273)				10-13 years (*n* = 4485)				14-17 years (*n* = 4496)			
			Boy		Girl		Boy		Girl		Boy		Girl	
	n (%)	(95%CI)	n (%)	(95%CI)	n (%)	(95%CI)	n (%)	(95%CI)	n (%)	(95%CI)	n (%)	(95%CI)	n (%)	(95%CI)
Bangkok^a, b, c^	310 (26.7)	(24.2-29.3)	63 (37.5)	(30.5-45.0)	30 (18.8)	(13.5-25.5)	62 (32.8)	(26.5-39.8)	34 (18.7)	(13.7-25.0)	70 (33.3)	(27.3-40.0)	51 (20.1)	(15.6-25.4)
Central^c^	295 (25.2)	(22.8-27.8)	55 (28.2)	(22.4-34.9)	39 (21.4)	(16.1-28.0)	63 (30.1)	(24.3-36.7)	50 (25.6)	(20.0-32.2)	49 (28.5)	(22.3-35.7)	39 (18.1)	(13.5-25.4)
East^b, c^	193 (18.6)	(16.4-21.1)	38 (21.3)	(16.0-27.9)	25 (15.2)	(10.5-21.4)	55 (29.9)	(23.7-36.9)	23 (13.3)	(9.0-19.2)	34 (22.2)	(16.4-29.4)	18 (9.8)	(6.3-23.7)
West	161 (16.0)	(13.9-18.4)	35 (20.2)	(14.9-26.8)	26 (16.1)	(11.3-22.6)	35 (19.7)	(14.5-26.1)	20 (11.8)	(7.8-17.6)	24 (16.3)	(11.2-23.1)	21 (11.9)	(7.9-15.0)
North^a, b, c^	474 (20.7)	(19.1-22.4)	112 (30.5)	(26.0-35.4)	43 (12.6)	(9.5-16.5)	127 (32.4)	(28.0-37.2)	53 (14.3)	(11.1-18.3)	103 (27.8)	(23.5-32.6)	36 (8.0)	(5.9-17.6)
South^a, b, c^	477 (25.0)	(23.1-27.0)	131 (34.4)	(29.8-39.3)	87 (24.9)	(20.7-29.7)	96 (28.9)	(24.3-34.0)	64 (20.3)	(16.2-25.1)	63 (27.6)	(22.2-33.8)	36 (11.9)	(8.7-10.9)
North East^a, b, c^	1185 (25.3)	(24.1-26.6)	211 (28.2)	(25.1-31.5)	149 (21.2)	(18.3-24.3)	314 (38.1)	(34.8-41.4)	185 (24.0)	(21.1-27.1)	224 (30.7)	(27.5-34.1)	102 (11.3)	(9.4-16.0)
Total	3095 (234)	(22.6-24.1)	645 (29.2)	(27.3-31.1)	399 (19.3)	(17.7-21.1)	752 (32.6)	(30.7-34.5)	429 (19.7)	(18.1-21.4)	567 (28.2)	(22.6-30.2)	303 (12.2)	(11.0-13.5)

*The total count is different from Table 1, due to rounded cell counts. ^a^=Significant difference between genders for ages 6-9 years at *p* < 0.05. ^b^=Significant difference between genders for ages 10-13 years at *p* < 0.05. ^c^=Significant difference between genders for ages 14-17 years at *p* < 0.05

Analysis of PA by domain (Table 3) revealed that overall 92% of Thai children and youth participated in PE. Almost half (47.6%) engaged in organized sports and there was a consistently higher proportion of boys compared with girls across all school years. About half (52%) of children and youth reported usually using active transport to school, with the lowest proportion seen in the 6-9 year group. The majority of Thai children and youth participated in at least 3 sport/exercise and recreational activities. A high proportion of Thai children and youth also engaged in at least 3 screen-time activities across all age groups. Significant differences in participation were found between genders across all domains: PE, organized sports, recreational activities, and etc. (all *p* < 0.05). The achievement of the PA guideline was significantly correlated with children and youth who participated in organised sports, sports/exercise, and recreational activities (all *p* < 0.001), whereas no significant correlation was found with PE class, active travel to school, and screen-time activities.

**Table 3:** Prevalence of participation in different physical activity domains in Thai children and youth by age group and sex

	Total			6-9 years						10-13 years						14-17 years					
				Boy			Girl			Boy			Girl			Boy			Girl		
	n	%	(95%CI)	n	%	(95%CI)	n	%	(95%CI)	n	%	(95%CI)	n	%	(95%CI)	n	%	(95%CI)	n	%	(95%CI)
PE class†																					
Yes	12,181	91.9	(91.4-92.4)	1898	85.9	(84.4-87.3)	1782	86.4	(84.8-87.8)	2233	96.7	(95.9-97.4)	2097	96.4	(95.5-97.1)	1871	93.0	(91.8-94.1)	2300	92.6	(91.5-93.5)
Organized sports*†																					
Yes	6305	47.6	(46.7-48.4)	1051	47.5	(45.5-49.6)	899	43.6	(41.5-45.7)	1285	55.6	(53.6-57.6)	1122	51.6	(49.5-53.7)	1014	50.4	(48.2-52.6)	934	37.6	(35.7-39.5)
Active travel to school†																					
Yes	6885	51.9	(51.1-52.8)	1002	45.3	(43.3-47.4)	840	40.7	(38.6-42.9)	1425	61.7	(59.7-63.7)	1241	57.0	(54.9-59.1)	945	47.0	(44.8-49.2)	1432	57.6	(55.7-59.6)
Sport/exercise activities*†																					
None	520	3.9	(3.6-4.3)	34	1.5	(1.1-2.1)	58	2.8	(2.8-3.6)	30	1.3	(0.9-1.9)	44	2.0	(1.5-2.7)	109	5.4	(4.5-6.5)	245	9.9	(8.8-11.1)
1-2 activities	3016	22.8	(22.1-23.5)	319	14.4	(13.3-16.0)	389	18.9	(17.2-20.6)	300	13.0	(11.7-14.4)	395	18.2	(16.6-19.8)	621	30.9	(28.9-32.9)	992	39.9	(38.0-41.9)
3-4 activities	4074	30.7	(30.0-31.5)	564	25.5	(23.7-27.3)	584	28.3	(26.4-30.3)	684	29.6	(27.8-31.5)	692	31.8	(29.9-33.8)	741	36.8	(34.8-39.9)	809	32.6	(30.7-34.4)
≥ 5 activities	5645	42.6	(41.8-43.4)	1294	58.5	(56.5-60.6)	1031	50.5	(47.8-52.2)	1296	56.1	(54.1-58.1)	1045	48.0	(45.9-50.1)	540	26.9	(25.0-28.8)	439	17.7	(16.2-19.2)
Recreational activities*†																					
None	850	6.4	(6.0-6.8)	19	0.9	(0.6-1.3)	11	0.5	(0.3-1.0)	61	2.6	(2.1-3.4)	43	2.0	(1.5-2.7)	416	20.7	(19.0-22.5)	300	12.1	(10.9-13.4)
1-2 activities	3446	26.0	(25.3-26.8)	183	8.3	(7.2-9.5)	134	6.5	(5.5-7.6)	559	24.2	(22.5-26.0)	399	18.3	(16.8-20.0)	1008	50.1	(48.0-52.3)	1163	46.8	(44.9-48.8)
3-4 activities	3800	28.7	(27.9-29.5)	482	21.8	(20.1-23.6)	380	18.4	(16.8-20.1)	993	40.4	(38.4-42.4)	770	35.4	(33.4-37.4)	475	23.6	(21.8-25.5)	760	30.6	(28.8-32.4)
≥ 5 activities	5158	38.9	(38.1-39.8)	1527	69.1	(67.1-71.0)	1539	74.6	(72.6-76.4)	756	32.7	(30.9-34.7)	964	44.3	(42.2-46.4)	111	5.5	(4.6-6.6)	261	10.5	(9.4-11.8)
Screen-time activities†																					
None	189	1.4	(1.2-1.6)	23	1.0	(0.1-1.6)	31	1.5	(1.1-2.1)	43	1.9	(1.4-2.5)	27	1.2	(0.9-1.8)	35	1.7	(1.3-2.4)	30	1.2	(0.9-1.7)
1-2 activities	4515	34.1	(33.3-34.9)	906	41.0	(38.9-43.0)	1017	49.3	(47.1-51.5)	759	32.9	(31.0-34.8)	770	35.4	(33.4-37.4)	462	23.0	(21.2-24.9)	601	24.2	(22.6-25.9)
3-4 activities	6742	50.9	(50.0-51.7)	913	41.3	(39.3-43.3)	840	40.7	(38.6-42.9)	1207	52.3	(50.2-54.3)	1174	54.0	(51.9-56.1)	1082	53.8	(51.7-56.0)	1526	61.4	(59.5-63.3)
≥ 5 activities	1806	13.6	(13.1-14.2)	369	16.7	(15.2-18.3)	175	8.5	(7.4-9.8)	300	13.0	(11.7-14.4)	204	9.4	(8.2-10.7)	431	21.4	(19.7-23.3)	327	13.2	(11.9-14.6)

*Significantly associated with the achievement of the PA guideline at *p* < 0.001

†Significant difference between genders in all age groups at *p* < 0.05

Results from a multiple logistic regression analysis (Table 4) revealed that, when controlling for other demographic variables, girls were half as likely compared with boys to meet the PA guideline (OR = 0.49, *p* < 0.001) and children in the 10-13 age group had a 20% greater chance (OR = 1.20, *p* = 0.001) of achieving the PA guideline than their counterparts in the youngest age group. Children and youth who were overweight and obese were 16% (OR = 0.84, *p* = 0.03) and 22% (OR = 0.78, *p* = 0.01) respectively less likely to achieve the PA guideline compared with children and youth with normal weight. Children and youth who lived in regions other than Bangkok had significantly less likelihood (ranged from 17%-53%) of meeting the PA guideline, except the central region. The probability of meeting the PA guideline increased almost 40% (OR = 1.37, *p* < 0.001) in children and youth who participated in any organized sports when compared to those who did not. The probability increased more than twice when children and youth engaged in 3-4 sport/exercise activities (OR = 2.23, *p* < 0.001) and almost three times (OR = 2.93, *p* < 0.001) when they engaged in ≥5 activities compared with those who did not do any sport/exercise activity. Conversely, if children and youth engaged in a lower number of recreational activities, the probability of meeting the PA guideline was 19%-21% less than those who engaged in ≥5 activities. Religion, participation in PE, active transport, and screen-time activities were not found to be significant predictors of achieving sufficient levels of PA in Thai children and youth.

**Table 4:** Multivariate logistic regression model for explanatory variables associated with meeting the PA guideline

	% Meeting PA guideline	OR	95%CI for OR		*p*
			Lower	Upper	
Gender					
Boy	30.1	1			-
Girl	16.8	0.49	0.45	0.54	0.001*
Age group					
6-9 years old	24.4	1			-
10-13 years old	26.3	1.20	1.08	1.34	0.001*
14-17 years old	19.3	1.07	0.93	1.23	0.365
BMI					
Underweight	24.8	1.00	0.91	1.11	0.935
Normal	22.6	1			-
Overweight	20.2	0.84	0.71	0.99	0.033**
Obese	19.2	0.78	0.64	0.94	0.010**
Geographical region					
Bangkok	26.7	1			-
Central	25.2	0.92	0.76	1.11	0.371
East	18.6	0.54	0.43	0.66	0.001*
West	15.9	0.47	0.38	0.59	0.001*
North	20.7	0.64	0.54	0.76	0.001*
South	25.0	0.79	0.67	0.94	0.001*
North East	25.3	0.83	0.71	0.97	0.001*
Organized sports					
No	19.4	1			-
Yes	27.8	1.37	1.26	1.50	0.001*
Sport activities					
None	9.6	1			-
1-2 activities	16.0	1.63	1.19	2.22	0.002*
3-4 activities	22.4	2.23	1.63	3.04	0.001*
≥ 5 activities	29.2	2.93	2.14	4.01	0.001*
Recreational activities					
None	23.3	1.16	0.93	1.44	0.186
1-2 activities	19.0	0.79	0.68	0.90	0.001*
3-4 activities	22.1	0.81	0.72	0.91	0.001*
≥ 5 activities	27.2	1			-

*p < 0.01, **p < 0.05

Reference category, Gender: Boy, Age group: 6-9 years old, BMI: Normal, Geographical region: Bangkok, Organized sport: no participation, Sport activities: None, and Recreational activities: ≥5 activities

*OR* Odds ratios, *CI* Confidence Interval, *p p*-value

## Discussion

This study investigated the prevalence of PA, children’s participation in different PA domains, and correlates associated with achievement of the WHO PA guideline in a nationally representative sample of Thai children and youth aged 6-17 years. It was found that although the majority of Thai children and youth reported their participation in many PA domains, a minority of them accumulated sufficient time spending in those activities to meet the PA guideline. Overall, the prevalence estimate of Thai children and youth who met the PA guideline was low (23.4%) compared with their Western counterparts, but slightly better than the average estimates at ASEAN (19.6%) [36] and global levels (19.7%) [7].

This study found that the correlates of PA in Thai children and youth, specifically sex and age, were consistent with international trends [15–18, 20]. Thai girls were much less likely to meet the PA guideline than boys, and particularly in the oldest age group. The results revealed that the prevalence of PA in Thai children and youth decreased with age with the lowest prevalence observed in the 14-17 year group. Interestingly, the prevalence in the 10-13 year group was slightly higher than the other 2 year groups and this finding might partially reflect the children’s transition from primary to secondary school at around 11-12 years of age. The disruption to early pattern of PA as children move from primary to secondary (high) school has been seen in other countries [19]. In Thailand it is common for children to move to a new school and most secondary schools are located in the city district. Consequently, children may have to travel far from home, which is reflected in the high proportion of children using active transport to attend school particularly in the 10-13 year group (Table 3). Use of active transport can contribute to a higher chance of meeting the PA guideline in the 10-13 year group [37], although in this study active transport was not sustained in the 14-17 year group. It is possible that the decline in the PA prevalence in the oldest age group might be due to the popular use of motorcycles among teenagers, and social values placed on high academic achievement [14].

Despite the reported inconclusive relationship between BMI and PA in the literature [22, 23], this study found that children’s BMI had a significant inverse relationship with meeting the PA guideline. This finding is similar to a study also conducted with a representative sample in the U.S.A [21] and many countries in Europe [22]. Although we cannot assert causality of the findings from our cross-sectional study, there would be no harm to suggest that efforts to increase participation in PA in Thai children and youth could be implemented concurrently with programs aimed at the prevention of weight status outside the normal BMI range.

Residential area was found to be significantly associated with children and youth’s participation in PA. When compared with Bangkok, children and youth living in other regions (except the central region) were significantly less likely to meet the PA guideline. These results are not unexpected given regional differences in terms of climate, economy, urbanisation, lifestyle, and culture. For example, the North Eastern Region has 10 times the number of people living in poverty compared with Bangkok [38] and may explain in part that children from the North East were significantly less likely to achieve sufficient PA level when compared to Bangkok children. Bangkok with a population of 10 million and high population density (4300 people/km^2^), is highly urbanised and accounts for almost 80% of the total urban area in the country [39]. Bangkok resident’s monthly household income is 2-5 times more than people living in other regions and their average monthly expenditure on recreational equipment and sports is 1.5-2.5 times more than people in other regions [40]. As a result, PA participation levels in Bangkok may be driven by these factors and therefore explain the difference compared with other areas. There are few international data available to compare the impact of the regional differences and levels of PA.

In addition to the socio-demographic factors, participation in organized sports consistent with previous studies [17, 24] was associated with achieving the PA recommended levels across genders and age groups. Almost half (47.6%) of the students reported that they participated in sport competitions/events organized by school or community (apart from PE) in the past academic year. Despite the absence of data on frequency and duration of the participation in organized sports, an interpretation for a minimum of participation at least once a year is associated with increased chance for children to meet sufficient PA level. The reported prevalence estimate of organized sport participation indicates that are still plenty of opportunities to improve levels of sports participation, particularly among girls in the oldest age group. Further study on the frequency and duration of participation in organized sports is needed to understand its contribution to meeting the PA guideline.

Participation in sport/exercise and recreational activities are another important PA domains found to have a strongly positive relationship with meeting the PA guideline in Thai children and youth. Our results support findings from other countries which show that participation in a number of sport/exercise and recreational activities declined with age [41]. This suggests that efforts to promote PA is needed on a continuous basis and sustain participation across the transition years of adolescence and ensure enjoyment and lifelong participation in physical activity. Furthermore, to meet the PA guideline, children and youth should be encouraged to engage in the sport/exercise and recreational activities as many as possible as our results showed the number of activities to be associated with meeting the recommendations. These results support one strategy outlined in the draft National PA plan which aimed to promote a wide variety of PA for enjoyment among 6-24 year-old population [42].

Interestingly and in contrast with other studies [24], we found that PE was not a significant predictor in achieving sufficient levels of PA, although almost all Thai children and youth participated in PE (92%). PE class is compulsory in Thailand but PE class time is at the school’s discretion based on the Ministry of Education’s suggested curriculum. There is no information on the proportion of class time allocated to PA that would contribute towards meeting PA recommendations. Our findings on participation in active transport also contrast with existing literature which typically reports a positive correlation between active transport and meeting PA guideline [37, 43]. Although half of Thai children and youth (52%) indicated that they usually used active means of transport to travel to and from school (Table 3), this was not a predictor of meeting the PA guideline. Further research is needed to better understand contribution of active transport to PA, particularly walking and cycling to and from school in Thailand.

Increasing sedentary behaviour is a worldwide concern for adults and children [7]. Our study found that Thai children and youth engaged in a large number of screen time activities but these activities were not a predictor of PA. Non-significant relationships between the number of screen time activities and PA found in this study supported conclusions from previous reviews [24]. It is possible that the number of activities children and youth engaged did not influence their PA and further study on frequency and amount of time is necessary to determine impact on achieving PA guideline.

Major strength of this study is the use of a previously validated instrument which not only allowed for international comparison but also culturally applicable examples of PA relevant to Thai children and youth. Strengths also include the use of the national representative sample covering a wide age range. Despite the strengths, some limitations need to be taken into account when interpreting the results. First, the PA prevalence estimates were based on a self-report instrument which might necessarily not measure the actual activities children and youth did. Recall ability and item misinterpretation are potential weaknesses of the self-reported instrument, especially in the youngest age group [44]. To increase data validity, this study used well-trained staffs to interview 6-9-year old children [33]. Second, the PA prevalence estimate could be influenced by seasonality. However, in our case, we considered that climatological data was not helpful in explaining the prevalence. The narrowly varied temperature, monthly average rainfall in all regions, and rain patterns were unlikely to explain the PA prevalence estimate. Third, due to the aim of this study to cover a wide age range and young children’s limited recall ability, we did not collect frequency and duration of children’ s participation in different PA domains across all age groups. Fourth, different time scales were used to measure children’s participation in the different PA domains (i.e. “in the past 7 days” for sports/exercise and recreation, the “current semester” for PE class, and in the “past academic year” for organized sports) to accommodate respondent burden and Thai local contexts. Finally, interpretation of the results on predictors of achieving PA guideline in Thai children and youth should be done with caution as the cross-sectional study design of this study does not allow for a causal relationship.

## Conclusion

This is the first nationwide study which investigates Thai children and youth’s participation in PA and its correlates in a representative sample. The results showed that two thirds of Thai children and youth were not sufficiently active. Thai children and youth did engage in a large number of PA but the prevalence estimate of meeting the PA recommendations was low. The results from this study support the development of the National PA plan by suggesting program interventions aimed at increasing PA levels in children and youth across all socio-demographic characteristics with special attention to girls, children with weight status outside the normal BMI range and living in regional areas. Children and youth’s high engagement in various PA domains provides a good opportunity to promote all possible types of PA as part of Thai children and youth’s daily life.

## Abbreviations

BMI: Body Mass Index
CI: Confidence interval
Df: Degrees of Freedom
OR: Odds ratio
PA: Physical activity (or activities)
PE: Physical Education
WHO: World Health Organization
